# Structural and Optical Characterization of Mechanochemically Synthesized CuSbS_2_ Compounds

**DOI:** 10.3390/ma15113842

**Published:** 2022-05-27

**Authors:** Luís Esperto, Isabel Figueira, João Mascarenhas, Teresa P. Silva, José B. Correia, Filipe Neves

**Affiliations:** 1 LNEG, Laboratório Nacional de Energia e Geologia, Estrada do Paço do Lumiar 22, 1649-038 Lisboa, Portugal; luis.esperto@lneg.pt (L.E.); isabel.figueira@lneg.pt (I.F.); joao.mascarenhas@lneg.pt (J.M.); brito.correia@lneg.pt (J.B.C.); 2 LNEG, Laboratório Nacional de Energia e Geologia, Estrada da Portela, Bairro do Zambujal—Alfragide, Apartado 7586, 2610-999 Amadora, Portugal; teresa.pena@lneg.pt

**Keywords:** powder technology, mechanochemical synthesis, absorber materials, chalcostibite

## Abstract

One of the areas of research on materials for thin-film solar cells focuses on replacing In and Ga with more earth-abundant elements. In that respect, chalcostibite (CuSbS_2_) is being considered as a promising environmentally friendly and cost-effective photovoltaic absorber material. In the present work, single CuSbS_2_ phase was synthesized directly by a short-duration (2 h) mechanochemical-synthesis step starting from mixtures of elemental powders. X-ray diffraction analysis of the synthesized CuSbS_2_ powders revealed a good agreement with the orthorhombic chalcostibite phase, space group Pnma, and a crystallite size of 26 nm. Particle-size characterization revealed a multimodal distribution with a median diameter ranging from of 2.93 μm to 3.10 μm. The thermal stability of the synthesized CuSbS_2_ powders was evaluated by thermogravimetry and differential thermal analysis. No phase change was observed by heat-treating the mechanochemically synthesized powders at 350 °C for 24 h. By UV-VIS-NIR spectroscopy the optical band gap was determined to be 1.41 eV, suggesting that the mechanochemically synthesized CuSbS_2_ can be considered suitable to be used as absorber materials. Overall, the results show that the mechanochemical process is a viable route for the synthesis of materials for photovoltaic applications.

## 1. Introduction

In general, photovoltaic (PV) technologies can be divided into two main categories: wafer-based and thin-film (TF) cells [1,2]. Presently, the technologies classified as commercial for terrestrial application include wafer-based crystalline silicon (c-Si) PV, as well as the TF technologies of CdTe and Cu(In,Ga)Se_2_ (CIGS) [1,2,3,4,5]. Additionally, the wafer-based c-Si PV technologies are divided as monocrystalline or multicrystalline [1,2,4,5]. Current market share is clearly dominated by the wafer-based c-Si PV technology, accounting for about 95% of the total production in 2020, with the share of monocrystalline technology now being about 84% of total c-Si production [4]. Consequently, the TF technology market share represents about 5% of today’s global annual market. In terms of solar-cell efficiencies, the record lab-cell efficiency is 26.7% for monocrystalline and 24.4% for multicrystalline wafer-based c-Si technology. The highest lab efficiency in TF technology is 23.4% for CIGS and 21.0% for CdTe solar cells [4]. Another emerging precommercial TF technology is hybrid organic–inorganic perovskite CH_3_NH_3_PbI_3_, which shows a remarkable record lab-cell efficiency of 25.5% [4]. However, to satisfy the expected world’s energy demand for the next decades, on the scale of tens of terawatt, these technologies are affected by many underlying issues, namely [1,5,6,7,8,9,10,11]: 95% of total c-Si PV module production is concentrated in the Far East, the capital intensity of Si may be difficult to reduce, the scarcity and increasing prices of materials (e.g., of In and Te), the use of materials in other energy-related technologies (batteries, power electronics, etc.), the environmental friendliness of Cd and Pb are questionable and the intrinsic instability of perovskite-based devices (due to environmental factors such as oxygen, moisture, thermal stress, light and applied electric fields).

Taking into account the above-mentioned considerations, several other criteria—besides efficiency and reliability—also become important and the research focus should be then directed towards solar cells based on absorber materials containing earth-abundant, low-cost and environmentally sustainable elements. Particularly, the development of In- and Te-free chalcogenides offer attractive options for the synthesis of absorber materials with appropriate bandgaps for photovoltaic conversion. One of those absorber materials is the quaternary kesterite Cu_2_ZnSnS_4_ (CZTS), and the related compounds Cu_2_ZnSnSe_4_ (CZTSe) and Cu_2_ZnSn(S,Se)_4_ (CZTSSe), in which the scarce elements in CIGS are replaced with the relatively abundant Zn and Sn [10,12,13]. Besides having similar optoelectronic properties with CIGS, CZTS shows a direct optical gap in a range suitable for photovoltaic applications. In 2013, the 12.6% record efficiency was demonstrated in CZTSSe [14]. However, further improvements are being hampered by compositional heterogeneities, complex defect chemistry, strong tendency for disorder and nonideal device architecture [10,13]. These limitations are leading to the research of absorber materials based on less complex chalcogenides compounds, such as the Cu–Sb–S (CAS)-based materials [10,11].

In the CAS system, the most promising phase known to have good prospect as absorber material for new single-junction or tandem TF solar cells is CuSbS_2_ (chalcostibite) because of its high optical-absorption coefficient (over 10^5^ cm^−1^), p-type electrical conductivity, tunable optical band gap (1.4–1.5 eV), low fabrication temperatures and a high value of spectroscopic limited maximum efficiency of 22.9% [11,15]. However, up to now the efficiencies of TF solar cells with CuSbS_2_ absorbers remain close to 3% [16]. A variety of physical and chemical methods have been used for the synthesis of CuSbS_2_ absorbers, including sputtering, electrodeposition, chemical bath deposition, spray pyrolysis deposition and advanced powder technologies [11,17,18,19,20,21,22,23,24,25,26,27,28]. Within them, the use mechanochemical synthesis (MCS) process, a solid-state synthesis route using high-energy ball mills, is being considered as a faster, scalable and environmentally friendly technology than the conventional processing routes for producing chalcogenides compounds [17,21,29,30,31]. In fact, the MCS process is charecterized by high levels of mechanical energy input, which gives rise to defects and structural changes that affect the chemical reactivity of the solids being processed, and consequently, the chemical reactions are activated in a fast way at ambient pressure and room-temperature conditions [29].

The present work describes experimental studies related with the direct synthesis of CuSbS_2_ by a short-duration MCS step starting from mixtures of elemental powders. The observed strong thermal stability of the structure and the determined optical bandgap of 1.41 eV allow us to infer the suitability of the MCS process for the production of PV materials and the potentialities of the synthesized materials as absorbers for TFSCs.

## 2. Materials and Methods

The CuSbS_2_ powders were synthesized by MCS performed on a Retsch high-energy planetary ball mill PM400 (Retsch GmbH, Haan, Germany). Mixtures of the elemental copper (Cu; Alfa Aesar (Haverhill, MA, USA), 99.9%, 10 μm), antimony (Sb; Sigma-Aldrich (affiliated of Merck KGaA, Darmstadt, Germany), 99.5%, <149 μm) and sulfur (S; Alfa Aesar (Haverhill, MA, USA), 99.999%, random sizes) powders, in the ratio of 1:1:2, were filled into 250 mL stainless-steel jars together with 26 balls with a diameter of 15 mm and without any additional fluid medium. These experiments were performed at a rotational speed of 340 rpm for a total milling duration of 2 h. Before starting the MCS, the jars were evacuated and back-filled with Argon. A small fraction of the MCS powders was heat-treated in vacuum (10^−2^ mbar) at 350 °C for 24 h using a conventional tube furnace.

Powder X-ray diffraction data was collected using a D8 Advance Bruker AXS diffractometer (Bruker AXS GmbH, Karlsruhe, Germany) with Cu Kα radiation. The XRD data treatment was performed by using DIFFRAC.EVA v5 software (Bruker AXS GmbH, Karlsruhe, Germany) [32], for phase identification, and DIFFRAC.TOPAS v6 software ((Bruker AXS GmbH, Karlsruhe, Germany)) [33], for a full-pattern Rietveld refinement [34]. As a measure for the goodness of the refinements the weighted profile R factor, R_wp_, has been used. Morphology and chemical elemental mapping of the powder particles were obtained, respectively, by scanning electron microscopy (SEM) and energy dispersive X-ray spectroscopy (EDS) using a Philips XL30 field-emission SEM ((FEI, Eindhoven, the Netherlands)) fitted with a Thermo Scientific™ UltraDry EDS detector (Thermo Fisher Scientific, Waltham, MA, USA). Particle-size distribution of the produced CuSbS_2_ powders was determined with a CILAS 1064 laser granulometer. Thermogravimetry (TG) and differential thermal analysis (DTA) were performed on a Setaram TG-DTA 92–16 thermobalance (SETARAM Instrumentation, Caluire, France) to evaluate the thermal stability of the synthesized powders from room temperature up to 600 °C. The TG-DTA experiments were carried out in specimens with masses of around 50 mg, with heating and cooling rates of 7 °C min^−1^ and under a high-purity Argon gas flow. Diffuse reflectance spectra were collected using a PerkinElmer Lambda 950 UV/Vis/NIR Spectrophotometer (PerkinElmer, Waltham, MA, USA), with InGaAs integrating sphere, in the spectral range of 350–1250 nm for band-gap energy assessment. Diffuse reflectance spectra were collected using PerkinElmer UV WinLab software in the spectral range of 350–1500 nm. The optical absorption spectra were converted from diffuse reflectance spectra using the Kubelka–Munk function [35,36]:

*F*(*R*) = *K*/*S* = (1 − *R*)^2^/2*R*, (1)

where *R* is the diffuse reflectance for an infinitely thick sample, *K* and *S* are the Kubelka–Munk absorption and scattering coefficients, respectively. In most cases, *S* can be considered as a constant independent of wavelength. In the parabolic band structure, the band gap (*Eg*) and absorption coefficient are related through the Tauc equation [35,36]:

*αhν* = *A_1_*(*hν*-*E_g_*)*^n^*, (2)

where *α* is the linear absorption coefficient of the material, *A_1_* is an arbitrary constant, *hν* is the photon energy, and *n* is constant depending on the band-gap nature: *n* = 1/2 for direct allowed transition band-gap materials and *n* = 2 for indirect allowed transition band-gap materials. Assuming the material scatters in perfectly diffuse manner, the Kubelka–Munk absorption coefficient *K* becomes equal to 2*α*. Thus, the relational expression becomes:

*F*(*R*)*hν* = *A_2_*(*hν*-*E_g_*)*^n^*. (3)

The *Eg* estimation was performed by extrapolating the linear region of the Tauc plot ((*F*(*R*)*hν*)^2^ versus *hν*) to the horizontal axis and considering the intersecting point.

## 3. Results and Discussion

**Figure 1:**
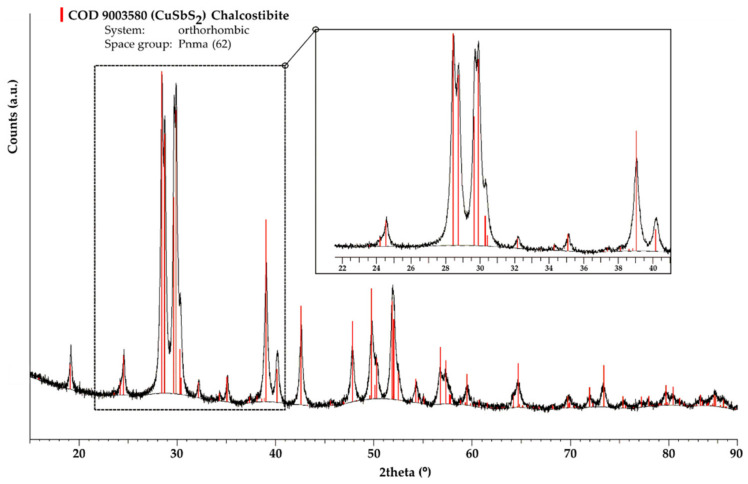
XRD pattern of CuSbS_2_ powders produced directly by mechanochemical synthesis for 2 h (solid black line—observed XRD pattern, thin black line—background).

Figure 1 shows the XRD pattern of the mechanochemically synthesized CuSbS_2_ powders. All the main reflections from the XRD pattern can be assigned to the CuSbS_2_ chalcostibite orthorhombic structure with the space group Pnma (62) (COD 9,003,580 [37]). Additionally, no secondary phases were detected, while the observable broadening of the Bragg peaks is mainly a consequence of the MCS process, which causes low crystallite size [38]. This result reflects the successful and fast conversion of the pristine elements into the chalcostibite phase with the experimental conditions used in the MCS. The reaction pathway leading to the direct synthesis of the chalcostibite CuSbS_2_ compound during the MCS process can be roughly assumed to follow the same path reported for other ternary chalcogenides [39,40]: (1) at the early stage of the MSC, the reaction between Cu and S occurs resulting in the formation of Cu-S binary compounds; (2) with the continuation of the MCS, Sb starts to be involved in the reaction with the Cu-S binary compounds, giving rise to the formation of the ternary chalcostibite CuSbS_2_ compound.

**Figure 2:**
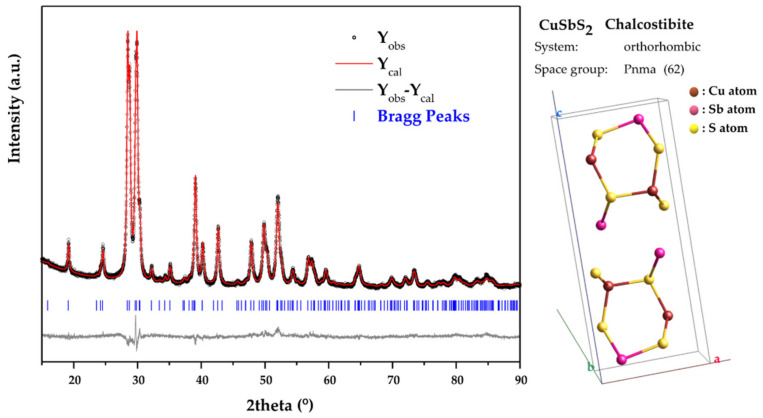
Rietveld refinement of the XRD pattern of CuSbS_2_ powders directly produced by mechanochemical synthesis.

Typical Rietveld analysis outputs for the 2 h MCS CuSbS_2_ powders are presented in Figure 2. The orthorhombic structure with the space group Pnma (62) mentioned above was used as structural model in the refinement. As it can be seen, there is a good match between the calculated XRD pattern (Y_cal_) and the observed XRD pattern (Y_obs_). This is corroborated by the values obtained for the R factors (R_exp_ = 2.56% and R_wp_ = 3.80%) which ensure the good level of the refinement. The obtained Rietveld refined structural lattice parameters are *a* = 6.0222(2) Å, *b* = 3.80317(16) Å and *c* = 14.5043(6) Å. These values are in good with the reference values for the chalcostibite orthorhombic structure from the COD 9,003,580 file (*a* = 6.018 Å, *b* = 3.7958 Å and c = 14.495 Å). Moreover, the crystallite size for the mechanochemically synthesized CuSbS_2_ powders was calculated to be 26.5 (4) nm.

**Figure 3:**
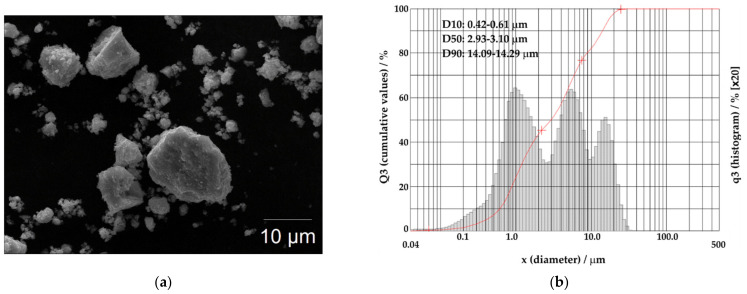
(**a**) SEM image and (**b**) cumulative particle-size distribution and histogram of particle-size distribution of the CuSbS_2_ powders directly produced by mechanochemical synthesis.

The morphology of the mechanochemically synthesized CuSbS_2_ powders is shown in Figure 3a. Based on SEM observations, the powder particles exhibit a quite irregular morphology, generically presenting three types of fractions: one at the submicrometric scale and the other two of the order of a few μm to tens of μm. In addition, it was seen that the submicron fraction tends to aggregate in micron-sized agglomerates. These observations were corroborated by the values of the characteristic dimensions of the particles (D10, D50 and D90) and by the granulometric distribution evaluated by laser diffractometry. As seen in Figure 3b, the frequency distribution curve (q3, histogram) revealed a multimodal distribution with three maxima. Moreover, the values obtained for D10, D50 and D90 means that 10% of the CuSbS_2_ powder particles are smaller than 0.52 µm, 50% are smaller than 3.02 µm, and 90% are smaller than 14.19 µm.

**Figure 4:**
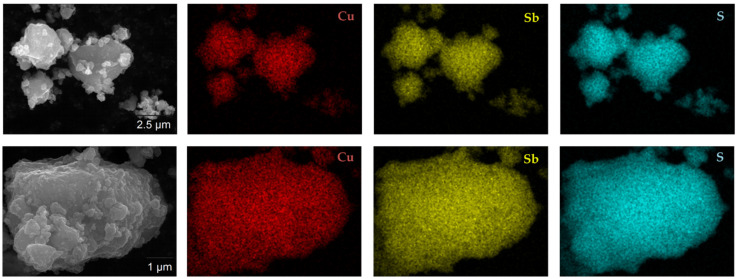
EDS elemental mapping for the CuSbS_2_ powder particles directly produced by mechanochemical synthesis.

EDS maps of the individual elements Cu, Sb and S allowed to address the degree of homogeneity of those elements within the produced CuSbS_2_ powder particles. As shown in Figure 4, all the elements are evenly distributed throughout the analyzed powder particles. Considering the starting elemental powder mixture, the uniform and homogeneous spatial distribution of Cu, Sb and S after the MCS process is extremely relevant.

**Figure 5:**
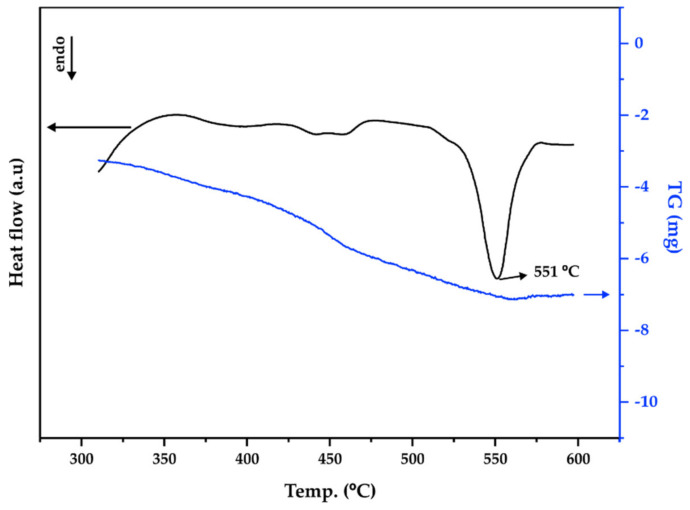
TG-DTA heating curves of mechanochemically synthesized CuSbS_2_ powders (DTA—black curve, TG—blue curve).

The DTA and TG heating curves of the mechanochemically synthesized CuSbS_2_ powders are shown in Figure 5. For temperatures above 400 °C, the DTA curve reveals two small endothermic (endo) peaks that are associated to the onset of the thermal decomposition of the chalcostibite CuSbS_2_ phase. According to the literature, the products of this thermal decomposition are Cu_12_Sb_4_S_13_, Sb_2_S_3_ and Sb_4_ [11,25]. The endothermic peak at 551 °C corresponds to the melting [11,25]. Furthermore, the small continuous weight loss observed in the TG curve, up to a temperature slightly above 551 °C, is clearly associated with the thermal decomposition of the chalcostibite CuSbS_2_ phase revealed by the DTA curve.

**Figure 6:**
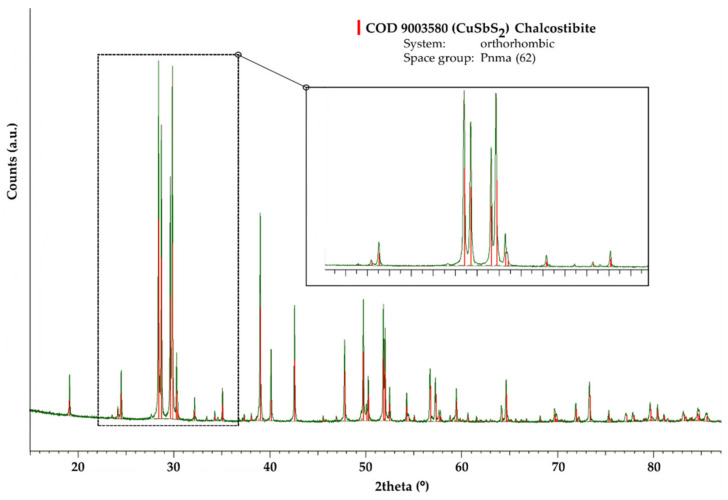
XRD pattern of mechanochemically synthesized CuSbS_2_ powders heat-treated at 350 °C/24 h (solid green line—observed XRD pattern, thin black line—background).

The thermal structural stability of the produced chalcostibite CuSbS_2_ phase was also addressed by XRD of the heat-treated mechanochemically synthesized CuSbS_2_ powders at 350 °C for 24 h in vacuum. It should be mentioned that the temperature chosen for this heat treatment was intentionally selected below the onset of the thermal decomposition of the chalcostibite CuSbS_2_ phase. As illustrated in Figure 6, all the main reflections from the obtained XRD pattern were again assigned to the orthorhombic structure with the space group Pnma (62). However, when compared to Figure 1, the observed Bragg peaks are now sharper and better defined. This can be attributed to the increase in the crystallite size and reduction in internal strains.

**Figure 7:**
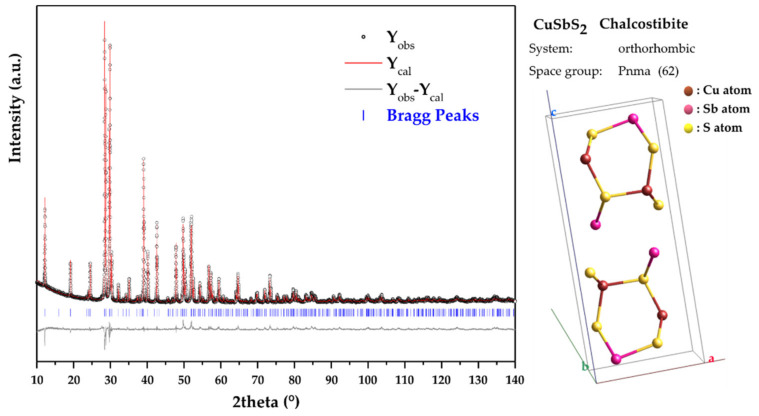
Rietveld refinement of the XRD pattern of mechanochemically synthesized CuSbS_2_ powders heat-treated at 350 °C/24 h.

Figure 7 shows the Rietveld analysis outputs for the heat-treated CuSbS_2_ powders. The values obtained for the R factors (R_exp_ = 2.29% and R_wp_ = 5.20%) confirm the good level of the refinement with the orthorhombic structure with the space group Pnma (62). The obtained Rietveld refined structural lattice parameters and the atomic coordinates of the constituent elements of Cu, Sb and S are listed in Table 1 and Table 2, respectively. As expected, these results put in evidence the recovery of the crystal structure due to the heat treatment, and as a consequence, the lattice parameters are closer to the standard values for the chalcostibite orthorhombic structure from the COD 9,003,580 file (Table 1) and in good agreement with the values reported in the literature [11]. Moreover, this was also supported by the crystallite size determined for the heat-treated CuSbS_2_ powders, which was calculated to be 141(5) nm, and consequently, greater than the crystallite-size value shown above (26.5(4) nm) for the mechanochemically synthesized powders.

**Figure 8:**
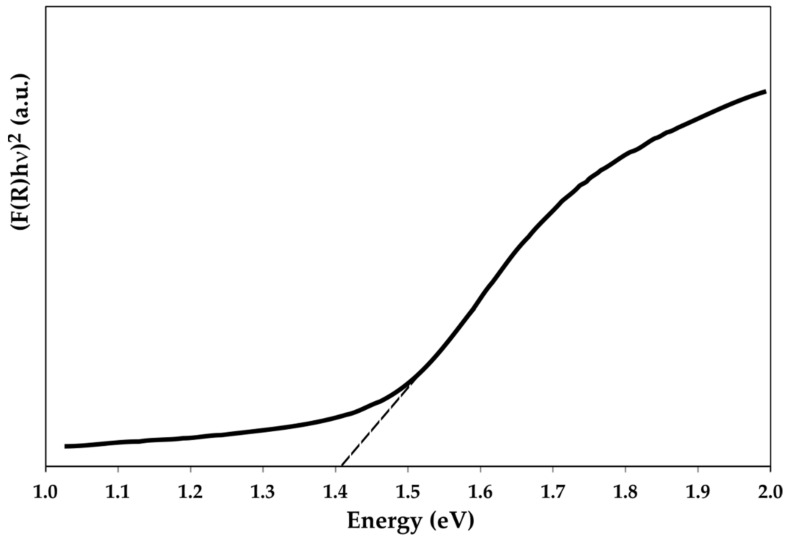
Tauc plot for the CuSbS_2_ compound directly produced by mechanochemical synthesis.

As shown in Figure 8, and explained in Section 2. Materials and Methods, the *Eg* estimation was performed by extrapolating the linear region of the Tauc plot to the horizontal axis and considering the intersecting point [35,36]. This has led to an *Eg* of 1.41 eV for the mechanochemically synthesized chalcostibite material, which is consistent with the theoretical and experimental values reported in the literature [11,15]. Consequently, the produced chalcostibite materials possess the expected optical characteristics. Considering that the optimal *Eg* should be in the range of 1.0–1.5 eV, the obtained result is very promising and can facilitate their potential application as an alternative absorber material for thin film solar cells. Thus, the ability to form thin films from the mechanochemically synthesized CuSbS_2_ powder, also using nonvacuum processes, is important for solar-cell fabrication and is being the subject of current studies.

## 4. Conclusions

Powders of CuSbS_2_, having orthorhombic structure with the space group Pnma (62), were synthesized directly through a short 2 h-duration mechanochemical step. The absence of any phase transformation with the heat treatment at 350 °C for 24 h, demonstrated the strong structural stability of the produced phase. The band-gap energy of the CuSbS_2_ powders was estimated by extrapolation to be of 1.41 eV, in good agreement with the values reported in the literature. The mechanochemically synthesized CuSbS_2_ compounds can then be considered suitable to be used as absorber materials for thin-film solar cells. Furthermore, the mechanochemical synthesis process proved to be a viable and promising route for the preparation of materials for photovoltaic applications.

## Figures and Tables

**Table 1:** Results of the Rietveld refinement for the lattice parameters of the mechanochemically synthesized CuSbS_2_ powders and of the heat-treated CuSbS_2_ powders. For comparison purposes, the standard values from the COD 9,003,580 file are also shown.

Lattice Parameter (Å)	MCS	Heat Treated	COD 9,003,580 File
*a*	6.0222 (2)	6.02061 (4)	6.018
*b*	3.80317 (16)	3.80171 (2)	3.7958
*c*	14.5043 (6)	14.50051 (10)	14.495

**Table 2:** Atomic coordinates (*x*, *y* and *z*) determined by the Rietveld refinement of the constituent elements of Cu, Sb and S of the heat-treated CuSbS_2_ powders.

Element	Site	Atomic Coordinates		
		*x*	*y*	*z*
Cu	4c	0.7522	0.2500	0.6724
Sb	4c	0.2260	0.2500	0.0633
S (1)	4c	0.6221	0.2500	0.0950
S (2)	4c	0.3706	0.2500	0.6756

## Data Availability

Data are contained within the article.

## References

[1] Zendehdel M., Nia N.Y., Yaghoubinia M., Gok A. (2020). Emerging Thin Film Solar Panels. Reliability and Ecological Aspects of Photovoltaic Modules.

[2] Jean J., Brown P.R., Jaffe R.L., Buonassisi T., Bulović V. (2015). Pathways for Solar Photovoltaics. Energy Environ. Sci..

[3] Taylor N., Jäger-Waldau A. (2020). Photovoltaics Technology Development Report 2020.

[4] (2022). Photovoltaics Report.

[5] Smith B.L., Woodhouse M., Horowitz K.A.W., Silverman T.J., Zuboy J., Margolis R.M. (2021). Photovoltaic (PV) Module Technologies: 2020 Benchmark Costs and Technology Evolution Framework Results.

[6] Divitini G., Cacovich S., Matteocci F., Cinà L., Di Carlo A., Ducati C. (2016). In Situ Observation of Heat-Induced Degradation of Perovskite Solar Cells. Nat. Energy.

[7] Feltrin A., Freundlich A. (2008). Material Considerations for Terawatt Level Deployment of Photovoltaics. Renew. Energy.

[8] Bae S., Kim S., Lee S.-W., Cho K.J., Park S., Lee S., Kang Y., Lee H.-S., Kim D. (2016). Electric-Field-Induced Degradation of Methylammonium Lead Iodide Perovskite Solar Cells. J. Phys. Chem. Lett..

[9] Mbumba M.T., Malouangou D.M., Tsiba J.M., Bai L., Yang Y., Guli M. (2021). Degradation Mechanism and Addressing Techniques of Thermal Instability in Halide Perovskite Solar Cells. Sol. Energy.

[10] Zakutayev A. (2017). Brief Review of Emerging Photovoltaic Absorbers. Curr. Opin. Green Sustain. Chem..

[11] Peccerillo E., Durose K. (2018). Copper—Antimony and Copper—Bismuth Chalcogenides—Research Opportunities and Review for Solar Photovoltaics. MRS Energy Sustain..

[12] Pal K., Singh P., Bhaduri A., Thapa K.B. (2019). Current Challenges and Future Prospects for a Highly Efficient (>20%) Kesterite CZTS Solar Cell: A Review. Sol. Energy Mater. Sol. Cells.

[13] Nazligul A.S., Wang M., Choy K.L. (2020). Recent Development in Earth-Abundant Kesterite Materials and Their Applications. Sustainability.

[14] Wang W., Winkler M.T., Gunawan O., Gokmen T., Todorov T.K., Zhu Y., Mitzi D.B. (2014). Device Characteristics of CZTSSe Thin-Film Solar Cells with 12.6% Efficiency. Adv. Energy Mater..

[15] De Souza Lucas F.W., Zakutayev A. (2018). Research Update: Emerging Chalcostibite Absorbers for Thin-Film Solar Cells. APL Mater..

[16] Banu S., Ahn S.J., Ahn S.K., Yoon K., Cho A. (2016). Fabrication and Characterization of Cost-Efficient CuSbS_2_ Thin Film Solar Cells Using Hybrid Inks. Sol. Energy Mater. Sol. Cells.

[17] Dutková E., Sayagués M.J., Fabián M., Kováč J., Kováč J., Baláž M., Stahorský M. (2021). Mechanochemical Synthesis of Ternary Chalcogenide Chalcostibite CuSbS_2_ and Its Characterization. J. Mater. Sci. Mater. Electron..

[18] Ornelas-Acosta R.E., Shaji S., Avellaneda D., Castillo G.A., Das Roy T.K., Krishnan B. (2015). Thin Films of Copper Antimony Sulfide: A Photovoltaic Absorber Material. Mater. Res. Bull..

[19] Wan L., Ma C., Hu K., Zhou R., Mao X., Pan S., Wong L.H., Xu J. (2016). Two-Stage Co-Evaporated CuSbS_2_ Thin Films for Solar Cells. J. Alloys Compd..

[20] Chalapathi U., Poornaprakash B., Ahn C.H., Park S.H. (2018). Two-Stage Processed CuSbS_2_ Thin Films for Photovoltaics: Effect of Cu/Sb Ratio. Ceram. Int..

[21] Zhang H., Xu Q., Tan G. (2016). Physical Preparation and Optical Properties of CuSbS_2_ Nanocrystals by Mechanical Alloying Process. Electron. Mater. Lett..

[22] Garza C., Shaji S., Arato A., Perez Tijerina E., Alan Castillo G., Das Roy T.K., Krishnan B. (2011). P-Type CuSbS_2_ Thin Films by Thermal Diffusion of Copper into Sb_2_S_3_. Sol. Energy Mater. Sol. Cells.

[23] Alqahtani T., Khan M.D., Lewis D.J., Zhong X.L., O’Brien P. (2021). Scalable Synthesis of Cu–Sb–S Phases from Reactive Melts of Metal Xanthates and Effect of Cationic Manipulation on Structural and Optical Properties. Sci. Rep..

[24] Cho A., Banu S., Kim K., Park J.H., Yun J.H., Cho J.S., Yoo J.S. (2017). Selective Thin Film Synthesis of Copper-Antimony-Sulfide Using Hybrid Ink. Sol. Energy.

[25] Welch A.W., Zawadzki P.P., Lany S., Wolden C.A., Zakutayev A. (2015). Self-Regulated Growth and Tunable Properties of CuSbS2 Solar Absorbers. Sol. Energy Mater. Sol. Cells.

[26] Edley M.E., Opasanont B., Conley J.T., Tran H., Smolin S.Y., Li S., Dillon A.D., Fafarman A.T., Baxter J.B. (2018). Solution Processed CuSbS_2_ Films for Solar Cell Applications. Thin Solid Films.

[27] Suehiro S., Horita K., Yuasa M., Tanaka T., Fujita K., Ishiwata Y., Shimanoe K., Kida T. (2015). Synthesis of Copper-Antimony-Sulfide Nanocrystals for Solution-Processed Solar Cells. Inorg. Chem..

[28] Septina W., Ikeda S., Iga Y., Harada T., Matsumura M. (2014). Thin Film Solar Cell Based on CuSbS_2_ Absorber Fabricated from an Electrochemically Deposited Metal Stack. Thin Solid Films.

[29] Neves F., Stark A., Schell N., Mendes M.J., Aguas H., Fortunato E., Martins R., Correia J.B., Joyce A. (2018). Investigation of Single Phase Cu_2_ZnSnxSb1-XS4 Compounds Processed by Mechanochemical Synthesis. Phys. Rev. Mater..

[30] Neves F., Correia J.B., Hanada K., Santos L.F., Gunder R., Schorr S. (2016). Structural Characterization of Cu_2_SnS_3_ and Cu_2_(Sn,Ge)S_3_ Compounds. J. Alloys Compd..

[31] Baláž P., Baláž M., Sayagués M.J., Eliyas A., Kostova N.G., Kaňuchová M., Dutková E., Zorkovská A. (2017). Chalcogenide Quaternary Cu_2_FeSnS_4_ Nanocrystals for Solar Cells: Explosive Character of Mechanochemical Synthesis and Environmental Challenge. Crystals.

[32] (2019). Bruker AXS DIFFRAC.EVA v5, Software for the Analysis of 1D and 2D X-Ray Datasets Including Visualization, Data Reduction, Phase Identification and Quantification, Statistical Evaluation. https://www.bruker.com/en/products-and-solutions/diffractometers-and-scattering-systems/x-ray-diffractometers/diffrac-suite-software/diffrac-eva.html.

[33] (2016). Bruker AXS DIFFRAC.TOPAS v6, Profile Fitting Based Software for Quantitative Phase Analysis, Microstructure Analysis and Crystal Structure Analysis. https://www.bruker.com/en/products-and-solutions/diffractometers-and-scattering-systems/x-ray-diffractometers/diffrac-suite-software/diffrac-topas.html.

[34] Rietveld H.M. (1969). A Profile Refinement Method for Nuclear and Magnetic Structures. J. Appl. Crystallogr..

[35] Beleanu A., Mondeshki M., Juan Q., Casper F., Felser C., Porcher F. (2011). Systematical, Experimental Investigations on LiMgZ (Z = P, As, Sb) Wide Band Gap Semiconductors. J. Phys. D. Appl. Phys..

[36] Viezbicke B.D., Patel S., Davis B.E., Birnie D.P. (2015). Evaluation of the Tauc Method for Optical Absorption Edge Determination: ZnO Thin Films as a Model System. Phys. Status Solidi Basic Res..

[37] Grazulis S., Chateigner D., Downs R.T., Yokochi A.F.T., Quiros M., Lutterotti L., Manakova E., Butkus J., Moeck P., Le Bail A. (2009). Crystallography Open Database—An Open-Access Collection of Crystal Structures. J. Appl. Crystallogr..

[38] Baláž P., Achimovicová M., Baláž M., Billik P., Zara C.Z., Criado J.M., Delogu F., Dutková E., Gaffet E., Gotor F.J. (2013). Hallmarks of Mechanochemistry: From Nanoparticles to Technology. Chem. Soc. Rev..

[39] Li J., Tan Q., Li J.F. (2013). Synthesis and Property Evaluation of CuFeS_2_-x as Earth-Abundant and Environmentally-Friendly Thermoelectric Materials. J. Alloys Compd..

[40] Zhang D., Yang J., Jiang Q., Fu L., Xiao Y., Luo Y., Zhou Z. (2016). Ternary CuSbSe_2_ Chalcostibite: Facile Synthesis, Electronic-Structure and Thermoelectric Performance Enhancement. J. Mater. Chem. A.

